# Simultaneous Electrospinning and Electrospraying for the Preparation of a Precursor Membrane Containing Hydrothermally Generated Biochar Particles to Produce the Value-Added Product of Carbon Nanofibrous Felt

**DOI:** 10.3390/polym13050676

**Published:** 2021-02-24

**Authors:** Xianfu Li, Tao Xu, Zhipeng Liang, Vinod S. Amar, Runzhou Huang, Bharath K. Maddipudi, Rajesh V. Shende, Hao Fong

**Affiliations:** 1 Department of Chemistry and Applied Biological Sciences, South Dakota School of Mines and Technology, Rapid City, SD 57701, USA; lixianfu@ahpu.edu.cn (X.L.); taoxu@hsph.harvard.edu (T.X.); zhipeng.liang@mines.sdsmt.edu (Z.L.); runzhou.huang@mines.sdsmt.edu (R.H.); 2 School of Chemical and Environmental Engineering, Anhui Polytechnic University, Wuhu 241000, China; 3 Department of Chemical and Biological Engineering, South Dakota School of Mines and Technology, Rapid City, SD 57701, USA; vinod.amar@sdsmt.edu (V.S.A.); bharathkiran.maddipudi@mines.sdsmt.edu (B.K.M.)

**Keywords:** electrospinning, electrospraying, biochar, carbon nanofibrous materials, energy storage and conversion

## Abstract

Biochar is a byproduct generated from the hydrothermal liquefaction of biomass, such as corn stover, in an anaerobic environment. This work aims to convert biochar into a value-added product of carbon nanofibrous felt. First, the biochar-containing precursor membrane was prepared from simultaneous electrospinning and electrospraying. After thermal stabilization in air and carbonization in argon, the obtained precursor membrane was converted into a mechanically flexible and robust carbon nanofibrous felt. Electrochemical results revealed that the biochar-derived carbon nanofibrous felt might be a good candidate as a supercapacitor electrode with a good rate capability and high kinetic performance.

## 1. Introduction

Electrospinning and electrospraying are similar electrohydrodynamic processes in which a suspension or solution can be spun or sprayed upon applying a high direct current (DC) voltage to generate fibers or particles, respectively [1]. In electrospinning, fibers are generated from a polymer solution/suspension that can produce a continuous jet/filament due to electric repulsion force [2]; in electrospraying, particles are made from the capillary breakup of jet/filament due to surface tension and low solution/suspension viscosity [3]. A simultaneous electrospinning and electrospraying technique is developed to produce a hybrid nanofibrous membrane with different types of nanofibers and particles by the simultaneous electrospinning and electrospraying of two spinnerets [4,5,6,7]. In addition, the traditional electrospinning and electrospraying can be modified by adding supercritical CO_2_ into the precursor polymeric solution, which can overcome some intrinsic limitations of the traditional processes—namely, surface tension and viscosity control [8]. The hybrid electrospun nanofibrous membrane consisting of two or more components could provide synergistic effects of the individual component to improve the overall properties [9,10]. Compared to making composites from composite dope, simultaneous electrospinning and electrospraying provides a more versatile approach to combine nanofibers with different types of particles with different properties. Particles might not be able to be incorporated into the polymer spinning dope for electrospinning due to their size, surface wettability, dispersibility, etc., which would result in poor nanofiber quality and even a failure to produce nanofibers. If the concentration of particles in the spinning dope is too high, it is also difficult to produce nanofibers of good quality. Additionally, with simultaneous electrospinning and electrospraying, the particles can be attached to the electrospun nanofiber surface without sacrificing the particles’ surface properties and functionalities.

Energy storage/conversion is an important application for hybrid membranes made by simultaneous electrospinning and electrospraying. The design of a freestanding binder-free electrode is crucial for the development of flexible energy storage/conversion devices with high electrochemical performance. Electrospraying alone can be adopted for the preparation of particles with a high capacity; however, the resulting particles need to be mixed with binder and carbon black and then coated onto the current collector, which reduces the overall electrode capacity. Through simultaneous electrospinning and electrospraying, a freestanding hybrid membrane/felt can be constructed in the form of particles enwrapped in nanofibers; such a structure is suitable for producing supercapacitor electrodes. For example, simultaneous electrospinning and electrospraying has been studied to produce a polyaniline/carbon nanofiber/particle supercapacitor electrode; this hybrid membrane electrode exhibited superior electrochemical performance [9]. In another example, a flexible 3D Si/C fiber paper electrode was synthesized by simultaneously electrospraying nano-Si-PAN (polyacrylonitrile) clusters and electrospinning PAN fibers, followed by carbonization. The combined technology allowed the uniform incorporation of Si nanoparticles into a carbon textile matrix to develop a nano-Si/carbon composite fiber paper [10].

It is known that biomass such as corn stover can be used to manufacture different types of bioproducts (e.g., bio-oils and C1 to C3 carboxylic acids) via hydrothermal liquefaction in an anaerobic environment; nevertheless, this process usually generates biochar as a byproduct, which is a carbon-rich porous solid in powder form [11]. The biochar acquired from the hydrothermal process is often considered as biowaste, despite the fact that it can be utilized for several applications, such as soil amendment and heat generation [12]. Therefore, it would be economically beneficial to utilize biochar particles to develop innovative carbon nanofibrous felt for high-value applications. Because of biochar’s carbon-rich feature, it is particularly promising for use in energy storage and conversion applications [13,14,15,16,17,18,19,20,21,22,23]. However, most of the reported biochar particles were in powder form rather than being incorporated in the matrix, which limits its application as a binder-free electrode.

In this work, the high-value application (i.e., supercapacitor electrode) of hydrothermally generated biochar was investigated. Specifically, the precursor membrane-containing biochar particles was first prepared by simultaneously electrospinning polyacrylonitrile (PAN) into nanofibers and electrospraying biochar particles; through the subsequent heat treatments (i.e., stabilization in the air followed by carbonization in argon), the obtained biochar-containing precursor membrane was converted into mechanically flexible/robust carbon nanofibrous felt. PAN was used as an example precursor polymer to produce carbon nanofibers, which provided a matrix/support for the biochar particles in the resulting hybrid carbon nanofibrous felt. The morphological structures of the membrane/felt before and after thermal treatments were characterized, and the application of carbon nanofibrous felt as a supercapacitor electrode was explored.

## 2. Materials and Methods

### 2.1. Materials

PAN (Mw = 150,000), poly(vinyl alcohol) (PVA, Mw = 13,000~23,000), *N*,*N*-dimethylformamide (DMF), ethanol, acetone, and Ni(NO_3_)_2_ were purchased from Sigma-Aldrich (St. Louis, MO, USA). The unhydrolyzed solids (UHS), which were derived from alkaline pretreated corn stover, underwent the hydrothermal liquefaction (HTL) process at 300 °C under the reactor pressure of 1600 psi to prepare the biochar samples. During the HTL process, Ni(NO_3_)_2_ was utilized as the catalyst and the UHS to deionized water ratio was set at 1:10. After the HTL process, the resulting biochar was rinsed with acetone to extract bio-oils and then treated at 400 °C for 2 h (under inert atmosphere) to remove volatile compounds and residual bio-oils retained after acetone extraction. Thereafter, the biochar powder was dispersed in ethanol and then added into a high-speed blender (i.e., a Waring Laboratory Blender). The biochar particle suspension was mechanically blended for 20 min and then transferred into a glass flask and dried for further use.

### 2.2. Apparatus Description and Methods

**Figure 1:**
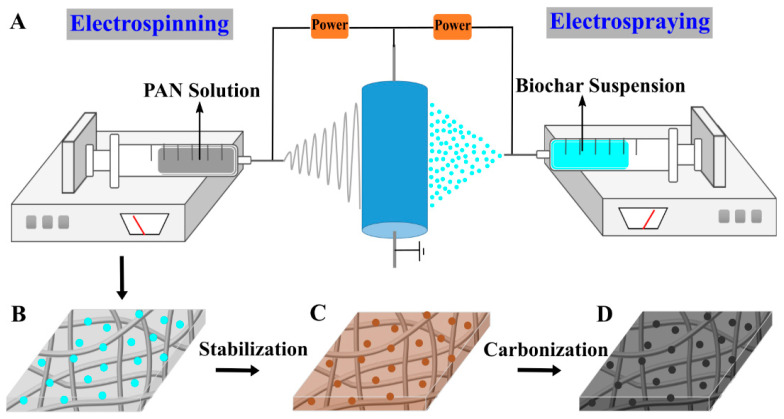
A schematic showing the preparation of carbon nanofibrous felt: (**A**) simultaneous electrospinning (**left**) and electrospraying (**right**), (**B**) polyacrylonitrile (PAN)/biochar precursor membrane, (**C**) stabilized membrane, and (**D**) carbon nanofibrous felt.

PAN was dissolved in DMF and mechanically stirred at room temperature for 12 h to prepare the electrospinning precursor solution (8.8 wt.%). Then, the solution was added into a BD Luer-Lok tip plastic syringe (30 mL) with a stainless steel needle (0.4 mm inner diameter and 90° blunt end). As illustrated in Figure 1A (left), the electrospinning system contained a laboratory-produced roller (Nanopareil, LLC, Rapid City, SD, USA) with a diameter of 25 cm and a high voltage power supply (Gamma High Voltage Research Inc., Ormond Beach, FL, USA). During the electrospinning process, 16 kV (a positive high DC voltage) was applied to the stainless steel needle, and the distance between the needle tip and the surface of an electrically grounded collector (i.e., the laboratory-produced roller covered with aluminum foil) was set at 25 cm. The rotational speed of the collector was set at 200 rpm, and the solution flow rate was set at 1.0 mL h^−1^ and controlled using a KDS 200 syringe pump (KD Scientific Inc., Holliston, MA, USA). The electrospraying setup was the same as the electrospinning setup (Figure 1A, right), but the suspension for electrospraying contained 5 g of fragmented biochar particles, 1.25 g of PVA, 200 mL of water, and 50 mL of ethanol. During electrospraying, the applied voltage was set at 12 kV and the flow rate was set at 1.0 mL h^−1^. Note that the mass fraction of PAN/biochar can be calculated by the flow rate and the PAN and biochar concentrations in each precursor solution. After calculation, the PAN/biochar ratio in this work was 3.6/1. PAN nanofibers and biochar particles were collected as randomly overlapped membrane on the roller (covered by aluminum foil), and this membrane was easily separated from the aluminum foil, as illustrated in Figure 1B. Subsequently, stabilization (Figure 1C) and carbonization (Figure 1D) were performed in a Lindberg 54,453 Heavy Duty Tube Furnace (TPS Co., Watertown, WI, USA). For the stabilization process, a constant flow of air was maintained through the furnace; a biochar/PAN nanofibrous membrane was sandwiched between two graphite plates (with the size of 10 cm × 5 cm), heated to 280 °C at the rate of 1 °C min^−1^, and then held at 280 °C for 6 h. For the carbonization process, a constant flow of argon with a flow rate of 100 mL/min was maintained through the furnace; the stabilized membrane was heated to 1200 °C at the rate of 5 °C min^−1^ and then held at 1200 °C for 2 h.

### 2.3. Characterization

Morphological structures of membrane/felt were examined by Zeiss Supra 40VP field-emission scanning electron microscopy (SEM) with a working distance of 4 mm, an electron high tension voltage of 1.5 kV, and a Type II secondary electrons (SE2) detector. The size of electrospun nanofibers was measured using the ImageJ software (*n* = 50). The crystalline structure of the electrospun carbon nanofibrous felt was investigated through X-ray diffraction (XRD) using a Bruker D8 Advance X-ray diffractometer, and the diffraction pattern was recorded at a scan rate of 4° min^−1^ in the 2θ range of 8–90°. A three-electrode system was used to study the electrochemical properties of carbon nanofibrous felt (as a supercapacitor electrode) at the room temperature of ~25 °C. In such a three-electrode system, the carbon nanofibrous felt was used as the working electrode, while platinum foil and a saturated calomel electrode (SCE) were used as the counter and reference electrodes, respectively. The electrochemical tests including cyclic voltammetry (CV) and galvanostatic charge-discharge (GCD) were carried out in 6 M KOH aqueous solution from −0.34 to 0.46 V (vs. SCE), and all the electrochemical studies were performed by utilizing an Autolab PGSTAT204 electrochemical workstation (Metrohm AG, Switzerland). The specific capacitance was calculated based on the equation *C* = (*I* × Δ*t*)/(*M* × Δ*V*), where *C* is the specific capacity (F g^−1^), *I* is the discharge current (A), Δ*t* is the discharge time (s), *M* is the mass of carbon nanofibrous felt in the working electrode (g), and Δ*V* is the potential range (V).

## 3. Results and Discussion

### 3.1. Fabrication of Biochar/PAN Carbon Nanofibrous Felt

Biochar alone cannot be electrospun/electrosprayed, owing to its particle morphology and lack of macromolecular entanglement/structure; on the contrary, PAN can be readily electrospun into uniform nanofibers with smooth surfaces from its solution. Upon simultaneous electrospinning and electrospraying, a hybrid biochar/PAN nanofibrous membrane can be acquired. Like electrospinning, a typical electrospraying setup consists of four main components, including a high-voltage supply, a metallic needle/spinneret, a syringe pump, and an electrically grounded collector. During this electrohydrodynamic process, surface charges are induced in the precursor solution inside the metallic needle by an external electrical field. The elongated precursor solution meniscus at the metallic needle tip forms a cone, and it is then extended at the apex and forms a permanent jet. Subsequently, the jet starts to break up into numerous charged particles, which can be self-dispersed well due to the Coulomb repulsion force. The Coulomb repulsion force can also prevent the charged particles from coagulation during the electrospraying. The electrospraying process can generate droplets/particles with sizes in the range from tens of microns to sub-microns [24]. Compared with other methods to combine particles with nanofibers, such as dip coating, these biochar particles can be uniformly embedded into carbon felt (among nanofibers) by simultaneous electrospinning and electrospraying, rather than only attached onto the nanofiber membrane surface. In addition, the number of particles can be readily controlled by tuning the particle concentration in the doping precursor and the electrospray flow rate.

**Figure 2:**
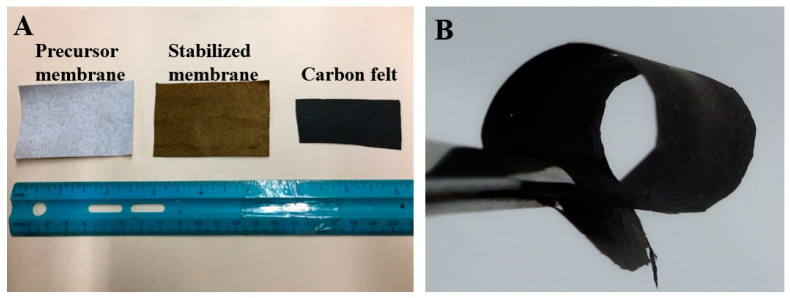
(**A**) A photo showing the precursor PAN/biochar nanofibrous membrane (**left**), stabilized membrane (**middle**), and carbon nanofibrous felt (**right**). (**B**) A photo showing the flexibility of the resulting carbon nanofibrous felt.

Through the electrospraying of the suspension consisting of biochar particles (with the sizes reduced to microns via mechanical fragmentation) and PVA (with the biochar/PVA mass ratio being 4/1), a relatively uniform biochar/PAN nanofibrous membrane was prepared, as shown in Figure 2A (left). It is noteworthy that the addition of PVA makes the biochar particle suspension stable enough for electrospraying; in other words, the suspension without PVA is not uniform/stable and thus cannot be continuously/steadily electrosprayed into biochar particles. Additionally, PVA is used as a binding agent to bind biochar particles with electrospun nanofibers during the thermal stabilization processing.

### 3.2. Morphology and Structure of Biochar/PAN Carbon Nanofibrous Felt

The acquired carbon nanofibrous felt maintained the overall morphology and structure of its precursor membrane, after the thermal treatments (i.e., stabilization and carbonization), as shown in Figure 2A (middle and right). The length and width of the membrane before and after carbonization were measured and the size reduction was calculated. The size of the membrane was reduced by about 50% due to the high-temperature carbonization processing. Intriguingly, the resulting carbon nanofibrous felt (Figure 2B) can be folded/bent without identifiable broken fibers or cracks, indicating its excellent mechanical flexibility and robustness; in contrast, the felt derived from PAN itself was much more brittle/fragile. The possible reason for this is that the biochar particles in the precursor membrane could mitigate the conglutination of PAN nanofibers, further making the prepared carbon nanofibrous felt fluffy; it is known that the carbon nanofibrous felt with low degree of fiber conglutination usually has a high mechanical flexibility/robustness. It is important to note that the freestanding characteristic and mechanical flexibility/robustness could make the biochar/PAN-based carbon nanofibrous felt suitable for a variety of applications, including wearable/portable devices and flexible electronics [25].

**Figure 3:**
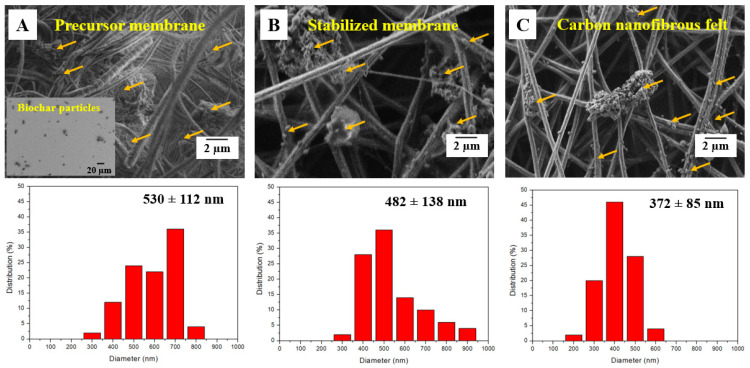
Scanning electron microscopy (SEM) images acquired from the (**A**) precursor membrane, (**B**) stabilized membrane, and (**C**) the resulting carbon nanofibrous felt, respectively, and their size distribution. The insert of (**A**) shows the SEM image of biochar particles. The arrows indicate the biochar particles that were enwrapped within the nanofibrous membrane/felt.

**Figure 4:**
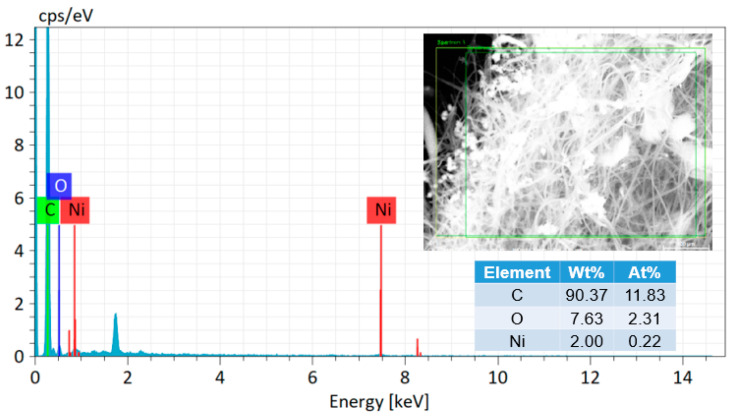
SEM and energy dispersive X-ray analysis (EDX) analysis of resulting carbon nanofibrous felt.

**Figure 5:**
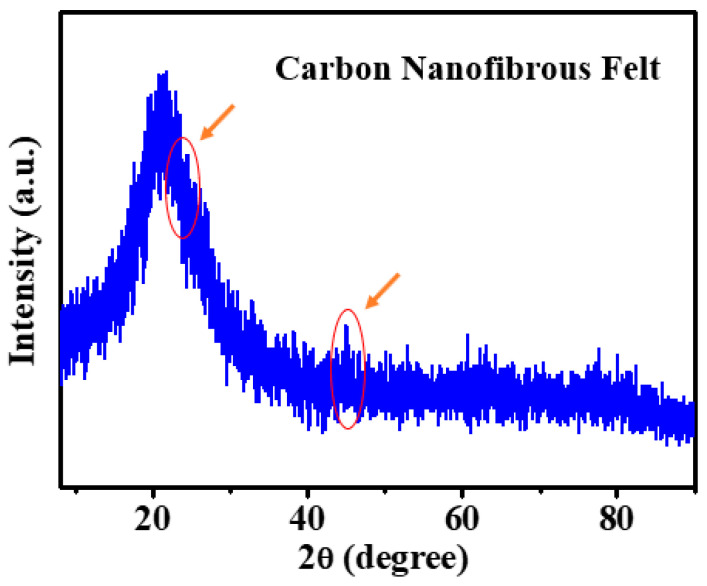
X-ray diffraction (XRD) pattern of the PAN/biochar carbon nanofibrous felt.

As exhibited in Figure 3A,B, the precursor membrane consisted of randomly overlaid PAN nanofibers and biochar particles; presumably, the particle surfaces were covered by PVA, which also acted as a binding agent between particles and nanofibers. The fiber diameter was reduced from 530 to 372 nm and carbonaceous structures were formed, which was attributed to the removal of heteroatoms (e.g., hydrogen, oxygen, and nitrogen) during the thermal treatments [26]. Figure 4 shows the SEM image with the energy dispersive X-ray analysis (EDX) of the resulting carbon nanofibrous felt. It is worth noting that Ni(NO_3_)_2_ was applied as the catalyst during the fabrication of biochar particles (i.e., the HTL process) and the residual catalyst could be converted into nickel oxide in carbon nanofibrous felt during the thermal treatment, which explains the presence of Ni element in the carbon felt. To investigate the crystalline structure of the resulting PAN/biochar carbon nanofibrous felt, XRD measurement was performed. As shown in Figure 5, the XRD diffractogram of the carbon felt displayed no obvious peaks of ordered graphitic structures, indicating the amorphous structure of carbon nanofibrous felt. [27]. Note that the peak around 2*θ* = 20° is from the SiO_2_ substrate that was used to hold the sample.

The purpose of this work is to turn biochar (which is a cheap biowaste) into a high-value application (i.e., supercapacitor electrodes). It is both economically and environmentally beneficial to utilize biochar particles to develop innovative carbon nanofibrous felt. In this study, PAN was used as an example precursor polymer to produce carbon nanofibers, which provided a matrix/support for biochar particles in the resulting hybrid carbon nanofibrous felt. In addition, any other precursor polymers such as polyimide, lignin, and cellulose can be used to produce the carbon nanofiber matrix to support biochar particles, which also demonstrates the versatility of such simultaneous electrospinning and electrospraying platforms.

### 3.3. Electrochemical Properties of Biochar/PAN Carbon Nanofibrous Felt

**Figure 6:**
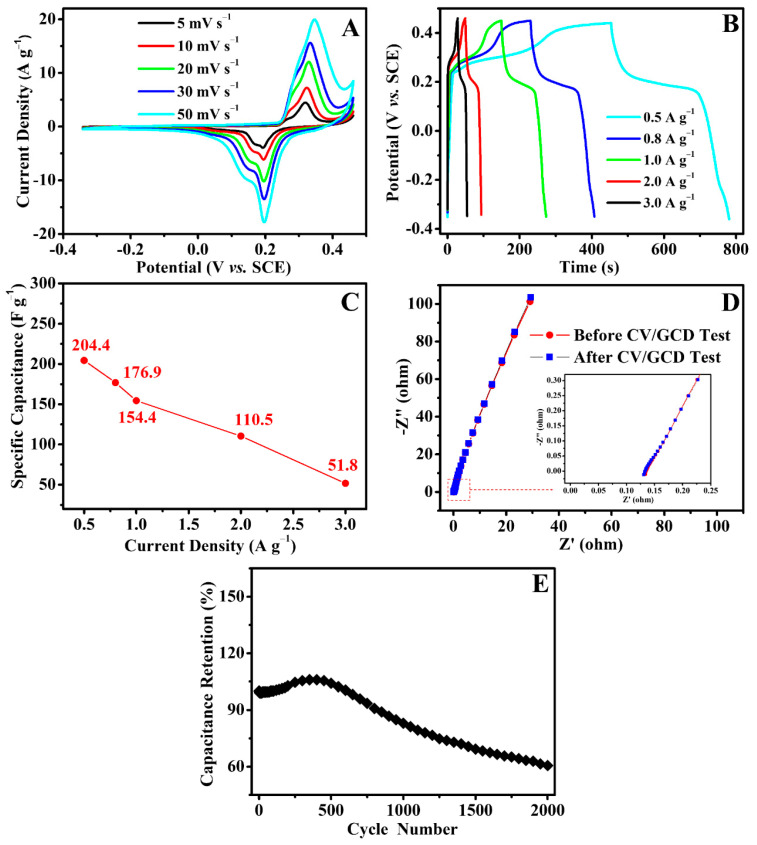
(**A**) CV profiles obtained from the carbon nanofibrous felt electrode at the scanning rates of 5, 10, 20, 30, and 50 mV s^−1^; (**B**) GCD curves of the electrode at different current densities; (**C**) specific capacitance values of the electrode at varied current densities; (**D**) Nyquist plots of the electrode before and after CV/GCD test; (**E**) cycling stability of the carbon nanofibrous felt electrode at 5 A g^−1^ 2000 times.

To investigate the electrochemical properties of the obtained carbon nanofibrous felt, CV and GCD tests were performed. Figure 6A shows the CV curves of the electrode made from carbon nanofibrous felt, and the scan rates were 5, 10, 20, 30, and 50 mV s^−1^, respectively. It is evident that all the electrodes show a pair of distinctive redox peaks, suggesting that the capacitance is primarily because of the pseudo-capacitive behavior of the electrode materials. The redox current was improved when the scan rate was increased. Additionally, the anodic peak shifts to the positive potential, and the cathodic peak shifts to the negative potential. The improving of the current response along with the scan rate suggests that the interfacial Faradic redox reaction kinetics and the electronic/ionic transport rate are fast enough under the different scan rates of 5, 10, 20, 30, and 50 mV s^−1^. Based on previous studies [28], electrospun carbon nanofibrous felt (derived from PAN under similar conditions/procedures) could only be directly utilized as double-layer capacitor electrodes rather than pseudo-capacitive electrodes, unless the carbon nanofibrous felt is surface decorated with electrocatalytically active materials (e.g., transition metal oxides such as MnO_2_). Consequently, the pseudo-capacitive behavior of the carbon felt obtained in this work must have been due to the nickel oxide from the biochar particles (Figure 4) which were embedded in the carbon membrane.

Figure 6B shows the GCD tests performed at various current densities. The specific capacitance values of this PAN/biochar-based carbon felt electrode at 0.5, 0.8, 1.0, 2.0, and 3.0 A g^−1^ were 204.4, 176.9, 154.4, 110.5, and 51.8 F g^−1^, respectively (Figure 6C). The capacitance retention of the electrode was 25.3% as the current density improved from 0.5 to 3.0 A g^−1^, suggesting that the rate capability of this electrode was further improved. The specific capacitance was gradually reduced with the increase in current density, possibly owing to the increase in voltage drop and the insufficient amount of active materials involved in the redox reaction. The carbon nanofibrous felt electrode was also evaluated by electrochemical impedance spectroscopy (EIS). Figure 6D shows the corresponding Nyquist plots. The arc in the high-frequency area found in each impedance plot was related to the charge transfer resistance (Rct) and contact resistance with the electrolyte (Rs), while the straight line at the low-frequency area was related to the Warburg impedance. It is important to note that the EIS profiles obtained from the carbon nanofibrous felt electrode before and after the CV and GCD tests were indistinguishable, suggesting that the biochar/PAN-based carbon electrode possessed very stable electrochemical properties. These results demonstrate excellent electrochemical performances. As shown in Figure 6E, the cycling test was carried out at 5 A g^−1^ 2000 times. Evidently, the specific capacitance retention of the carbon electrode gradually increased and then decreased, suggesting the electrode materials activation at the starting period of the GCD test. Specifically, the carbon electrode materials would be activated by the ion intercalation and de-intercalation, leading to the increment in active locations in the electrode materials; consequently, the specific capacitance was enhanced. The carbon nanofibrous felt electrode had a reasonably good cycling stability; after 2000 cycles, the electrode capacitance decreased to 39.4% of its initial value, demonstrating that the carbon nanofibrous felt electrode possessed long-term electrochemical stability.

## 4. Conclusions

In this work, the carbon nanofibrous felt was developed from a precursor membrane consisting of HTL-derived biochar particles and electrospun PAN nanofibers; its application as a supercapacitor electrode was investigated. The carbon nanofibrous felt was fabricated from the thermal stabilization and carbonization of PAN/biochar precursor membrane, which was prepared through simultaneous electrospinning and electrospraying. The PAN carbon nanofibers served as the matrix/support for the biochar particles. The electrochemical results revealed that the electrode capacitance was primarily due to the pseudo-capacitive behavior of carbon nanofibrous felt. Overall, the carbon nanofibrous felt electrode exhibited a good rate capability and high kinetic performance. This work demonstrates a high-value application of hydrothermally generated biochar; more importantly, it provides a simple but novel approach to developing carbon nanofibrous felt that can be used for a variety of applications, such as energy storage and conversion, catalyst support, and water purification.

## Data Availability

The data presented in this study are available on request from the corresponding author.
